# Dynamic microRNA Profiles of Hepatic Differentiated Human Umbilical Cord Lining-Derived Mesenchymal Stem Cells

**DOI:** 10.1371/journal.pone.0044737

**Published:** 2012-09-12

**Authors:** Lina Cui, Xinmin Zhou, Jinge Li, Liuyi Wang, Jingbo Wang, Qiang Li, Jindong Chu, Linhua Zheng, Qiong Wu, Zheyi Han, Yongquan Shi, Ying Han, Daiming Fan

**Affiliations:** 1 State Key Laboratory of Cancer Biology, Xijing Hospital of Digestive Diseases, The Fourth Military Medical University, Xi’an, Shaanxi Province, China; 2 Department of Gastroenterology, Yan’an University Affiliated Hospital, Yan’an, Shaanxi Province, China; University of Medicine and Dentistry of New Jersey, United States of America

## Abstract

Despite the extensive hepatic differentiation potential of human umbilical cord lining-derived mesenchymal stem cells (hUC-MSC), little is known about the molecular mechanisms of hUC-MSC differentiation. At the post-transcriptional level, microRNAs are key players in the control of cell fate determination during differentiation. In this study, we aimed to identify microRNAs involved in the hepatic differentiation of hUC-MSCs. After successfully isolating hUC- MSCs, we induced hepatocyte formation in vitro with growth factors. After 26 days of induction, hUC-MSCs could express hepatocyte-specific genes, synthesize urea and glycogen and uptake low-density lipoprotein. Cellular total RNA from hUC-MSCs and hepatic differentiated hUC-MSCs was collected at 7 time points, including 2 days, 6 days, 10 days, 14 days, 22 days and 26 days, for microRNA microarray analysis. Dynamic microRNA profiles were identified that did not overlap or only partially overlapped with microRNAs reported to be involved in human liver development, hepatocyte regeneration or hepatic differentiation of liver-derived progenitor cells. A total of 61 microRNAs among 1205 human and 144 human viral microRNAs displayed consistent changes and were altered at least 2-fold between hUC-MSCs and hepatic differentiated hUC-MSCs. Among these microRNAs, 25 were over-expressed; this over-expression occurred either gradually or increased sharply and was maintained at a high level. A total of 36 microRNAs were under-expressed, with an expression pattern similar to that of the over-expressed microRNAs. The expression of the altered expressed microRNAs was also confirmed by quantitative reverse-transcription polymerase chain reaction. We also found that microRNAs involved in hepatic differentiation were not enriched in hepatocyte or hepatocellular carcinoma cells and can potentially target liver-enriched transcription factors and genes. The elucidation of the microRNA profile during the hepatic differentiation of hUC-MSCs provides the basis for clarifying the role of microRNAs in hUC-MSC hepatic differentiation and specific microRNA selection for the conversion of hUC-MSCs to hepatocytes.

## Introduction

At the post-transcriptional level, microRNAs are emerging as key players in the control of proliferation and cell fate determination during differentiation. Studies have revealed that each type of cell differentiation is regulated by a specific microRNA. For example, adult neural stem/progenitor cell proliferation and neuronal differentiation is regulated by microRNA cluster miR-106b∼25 [1]. miR-150 controls B cell differentiation by targeting the transcription factor c-Myb [2]. miR-1 regulates smooth muscle cell differentiation by repressing Kruppel-like factor 4 [3]. miR-196a regulates proliferation and osteogenic differentiation in mesenchymal stem cells derived from human adipose tissue [4].

Moreover, microRNAs can also mediate cell transdifferentiation. Specific microRNAs can be used for cellular reprogramming. The expression of the miR-302/367 cluster can rapidly and efficiently reprogram mouse and human somatic cells to an iPSC state without requiring exogenous transcription factors [5], [6]. The expression of miR-9/9* and miR-124 in human fibroblasts can induce their conversion into neurons. The neurogenic transcription factors ASCL1 and MYT1L can enhance the rate of conversion and the maturation of the converted neurons, whereas expression of these transcription factors alone in the absence of miR-9/9*-124 is ineffective [7]. These studies indicate that one or several specific microRNAs can be used to convert adult cells derived from other sources into hepatocytes to efficiently obtain hepatocytes in vitro.

Mesenchymal stem cells (MSCs) possess plasticity and have the potential to differentiate into adipose tissue, bone, cartilage, tendon and muscle; thus, MSCs hold great hope for therapeutic applications. Adult bone marrow has been the most common source of MSCs for clinical applications. However, the supply of bone marrow is limited, and there is an age-dependent decrease in cell number. The umbilical cord and amniotic membrane are attractive sources of adult MSCs due to total global abundance, ease of culture, and fewer ethical concerns. Moreover, human umbilical cord-derived MSCs (hUC-MSCs) exhibit a more beneficial immunogenic profile and greater overall immunosuppressive potential than aged bone marrow-derived MSCs [8]. Like MSCs derived from bone marrow, hUC-MSCs can also be used to treat rat liver fibrosis [9] and improve glucose homeostasis in rats with liver cirrhosis [10].

HUC- MSCs can transdifferentiate into low immunogenic hepatocyte–like cells in conditioned culture medium [11], [12], [13]. However, little is known about the molecular mechanisms that regulate this progress, particularly the role of microRNAs. To further define the regulatory mechanisms of microRNAs, we examined the microRNA expression profile during HGF-induced hepatic differentiation of hUC-MSCs at seven different time points using microRNA microarrays and quantitative reverse transcription-polymerase chain reaction (qRT-PCR). A unique microRNA expression profile was associated with hUC-MSC hepatic differentiation; this profile was not enriched in hUC-MSCs or hepatocytes or hepatocellular carcinoma cells. The elucidation of the microRNA profile during hepatic differentiation of hUC-MSCs provides the basis for clarifying the role of microRNAs in hUC-MSC hepatic differentiation and specific microRNA selection for the conversion of hUC-MSCs into hepatocytes.

**Table 1:** Primers for target genes for qRT-PCR analysis.

**Target gene**	**Primer**	**Target gene**	**Primer**
CK-18	F: CCCTGCTGAACATCAAGGTCAA	miR-148a	TCAGTGCACTACAGAACTTTGT
	R: GCTGTCCAAGGCATCACCAA	miR-301a	CAGTGCAATAGTATTGTCAAAGC
HNF4α	F: AGCTGCAGATCGATGACAATGAG	miR-1290	TGGATTTTTGGATCAGGGA
	R: CATACTGGCGGTCGTTGATGTAG	miR-136	CAGCAGCAATTCATGTTTTGAA
ALB	F: ACTGCATTGCCGAAGTGGA	miR-424	CTTCCCCCCAGTAATCTTCATC
	R: GCAGCACGACAGAGTAATCAGGA	miR-30a	TGTAAACATCCTCGACTGGAAG
GAPDH	F:GCACCGTCAAGGCTGAGAAC	miR-31	AGGCAAGATGCTGGCATAGCT
	R:TGGTGAAGACGCCAGTGGA	miR-1246	AATGGATTTTTGGAGCAGG
miR-3646	AAAATGAAATGAGCCCAGCCCA	miR-100	AACCCGTAGATCCGAACTTGTG
miR-17*	ACTGCAGTGAAGGCACTTGTAG	miR-10a	TACCCTGTAGATCCGAATTTGTG
miR-3679-3p	CTTCCCCCCAGTAATCTTCATC	miR-130b	CAGTGCAATGATGAAAGGGCAT
miR-155	TTAATGCTAATCGTGATAGGGGT	miR-1973	ACCGTGCAAAGGTAGCATA
miR-146a	TGAGAACTGAATTCCATGGGTT	miR-29a	TAGCACCATCTGAAATCGGTTA
miR-671-5p	AGGAAGCCCTGGAGGGGCTGGAG	miR-31*	TGCTATGCCAACATATTGCCAT
miR-542-5p	TCGGGGATCATCATGTCACGAGA	miR-762	GGGGCTGGGGCCGGGGCCGAGC
miR-1185	AGAGGATACCCTTTGTATGTT	miR-17	CAAAGTGCTTACAGTGCAGGTAG
miR-539	GGAGAAATTATCCTTGGTGTGT	miR-542-3p	TGTGACAGATTGATAACTGAAA

## Materials and Methods

### Isolation and Culture of Human Umbilical Cord Lining-derived Mesenchymal Stem Cells

The present study was approved by the ethics committee of Xijing Hospital of The Fourth Military Medical University (Xi’an, China). Human umbilical cords were collected after full-term deliveries with informed written consent of the mothers (patient consent in Chinese/English and ethics statement from the ethics committee was shown in Supporting Information S1, S2, S3). HUC-MSCs were isolated as previously described [14]. Segments of the tissue was cut into pieces (∼1 inch long) and dissected to open the cord vessel. The pieces were placed in 250-mm plastic Petri dishes containing DMEM medium and incubated for approximately 1 day in a 5% CO2 incubator at 37°C. The Wharton’s jelly absorbed DMEM (containing phenol red) and was dissected with a razor; pieces of the outer envelope membrane were cultured after rinsing. Mesenchymal stem cell Expansion Medium (R&D Systems, Inc., Minneapolis, MN, USA) was used for the expansion of hUC-MSCs.

**Figure 1:**
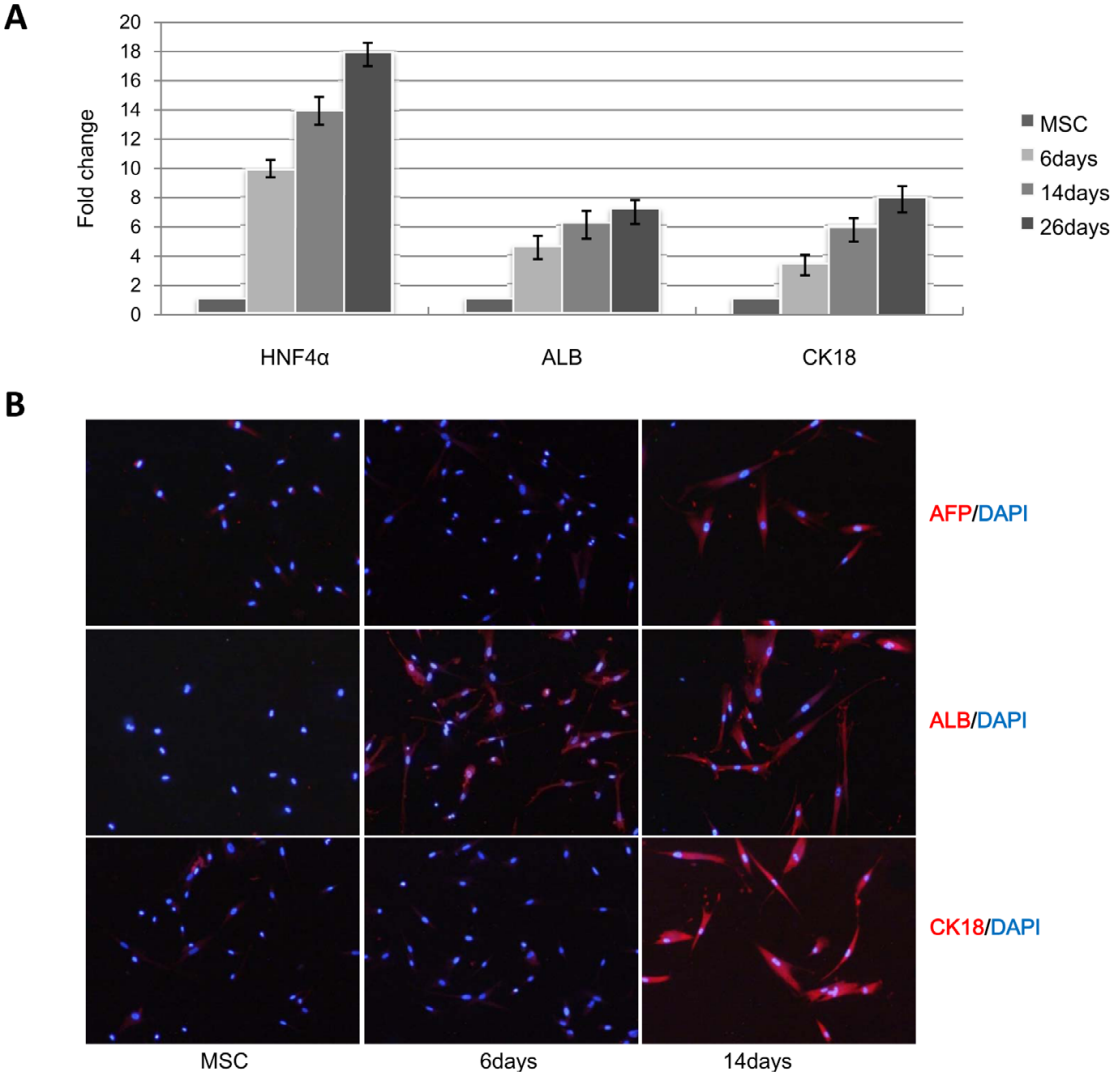
Hepatic differentiated hUC-MSCs can express hepatocyte–specific genes. A: Hepatocyte-specific gene expression analyzed by qRT-PCR in hUC-MSCs and hepatic differentiated hUC-MSCs for 6 days, 14 days and 26 days. Hepatocyte-specific gene expression was normalized to GAPDH expression, and the results are expressed relative to a value of 1 in the control hUC-MSCs. **B:** immunofluorescence of hepatocyte-specific gene expression in MSCs and hepatic differentiated MSCs. After induction for 14 days, hUC-MSCs can express AFP, ALB and CK18.

### Phenotypic Analysis

hUC-MSCs harvested at passage 3 (P3) were washed in phosphate-buffered saline (PBS) and incubated for 30 min with PBS containing 0.5% (w/v) bovine serum albumin and the following monoclonal antibodies: anti-human CD105-PE (eBioscience Inc., California, USA), anti-human CD31-FITC (BD Pharmingen Inc., San Diego, CA, USA), or anti-human CD34-FITC (eBioscience Inc.). After washing with PBS, the cells were analyzed with a Calibur flow cytometer (BD Pharmingen Inc.). The data were analyzed with Windows Multiple Document Interface for Flow Cytometry.

**Figure 2:**
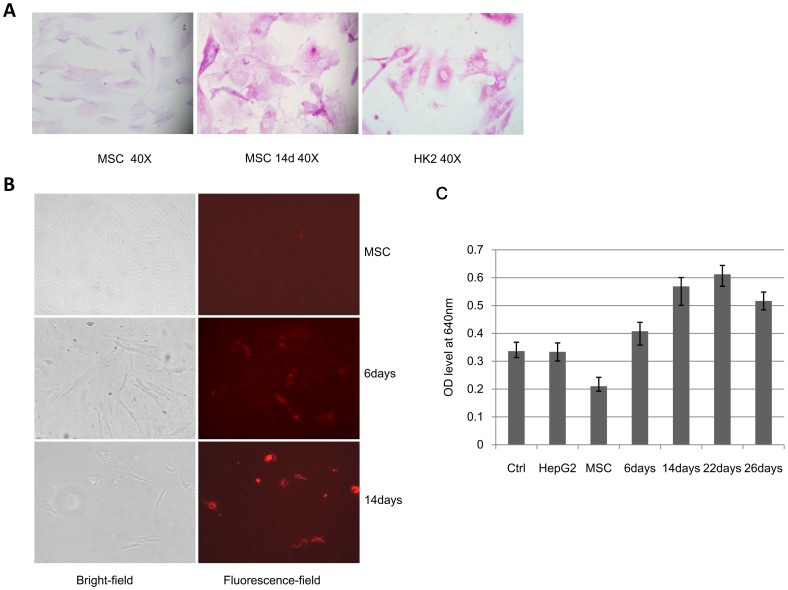
Hepatic differentiated hUC-MSCs possess hepatocyte-specific functions. A: PAS staining of hUC-MSCs before and after hepatic differentiation using HK2 as a positive control. **B:** Analysis of the LDL uptake ability of hepatic differentiated hUC-MSCs. **C:** Analysis of the BUN synthetic ability of hepatic differentiated hUC-MSCs.

### Differentiation Procedures

The differentiation potential of hUC-MSCs was examined using cells harvested at P3 to P5.

**Figure 3:**
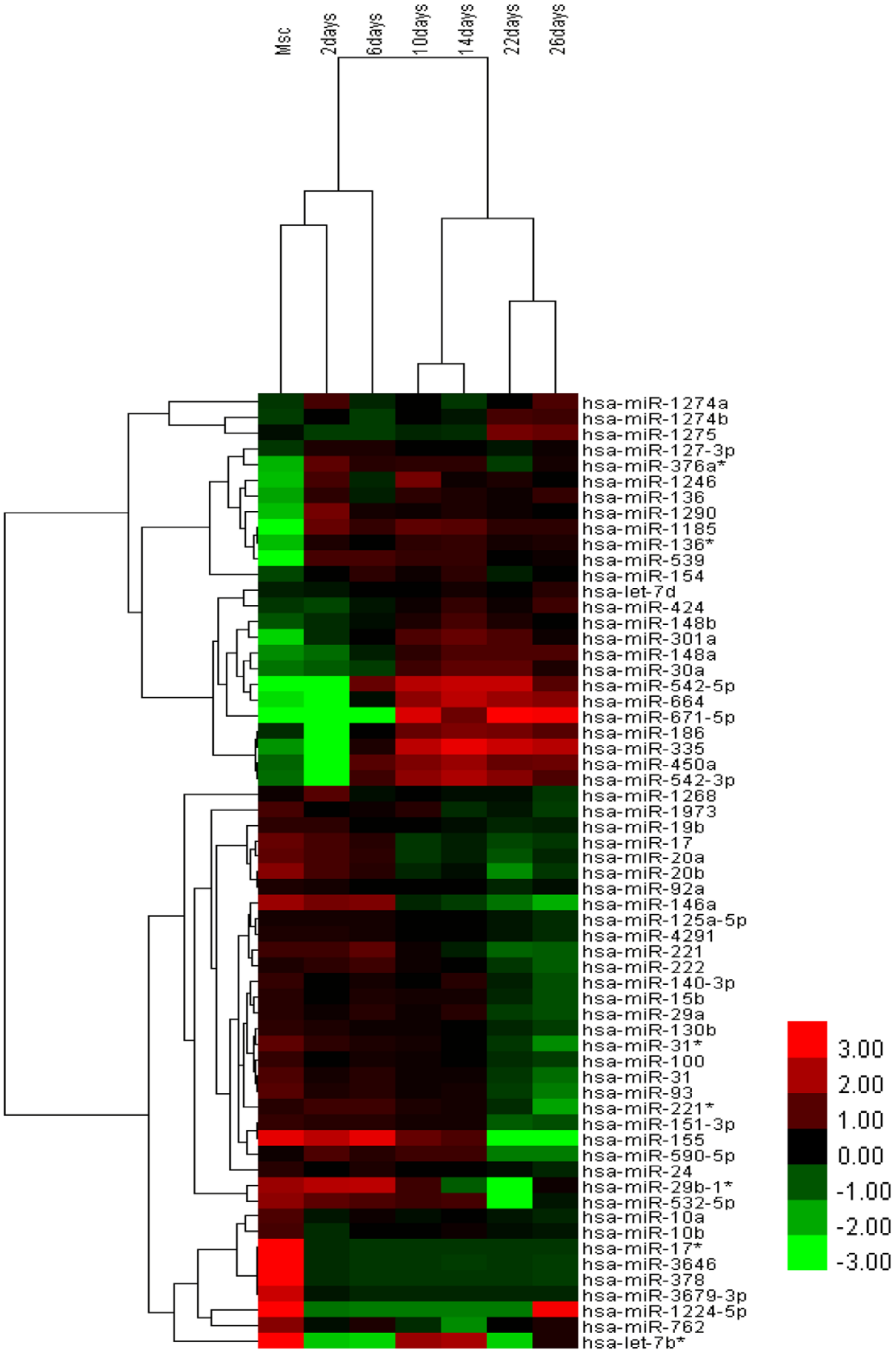
Heat map of 61 consistent altered expressed microRNAs. An unsupervised hierarchical cluster analysis was performed on differentially expressed microRNAs at seven time points during the hepatic differentiation of hUC-MSC (Cluster 3.0 software, average linkage). A dendogram, demonstrating the level of similarity in gene expression between the various samples and a heat map illustrating gene expression changes between the samples are shown. Samples are listed in columns, and microRNAs are listed in rows; a red color signifies high expression, while a green color signifies low expression, according to the color bar shown on the left in logarithmic scale.

#### Osteogenic differentiation

HUC-MSCs at P3 were plated in growth medium at a density of 1×10^4^ cells/cm^2^ in 6-well tissue culture plates that were pre-coated with fibronectin (FN) (Sigma-Aldrich Inc., St. Louis, Missouri, USA). After 24 hours, the growth medium was aspirated, and 2 ml of hUC-MSC osteogenic differentiation medium (Cyagen Bioscience Inc., Guangzhou, China) was added. After 3 weeks of differentiation, the cells were fixed with 2 ml 4% formaldehyde solution for 30 minutes, and the cells were stained with 1 ml alizarin red working solution for 4 minutes. Finally, the cells were visualized by light microscopy, and images were acquired.

**Figure 4:**
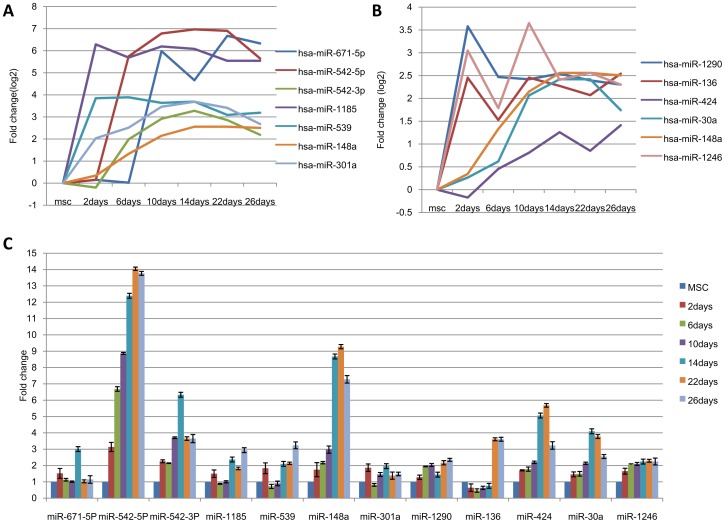
Over-expressed microRNA selection and analysis. **A:** The expression of seven microRNAs that changed ≥4-fold were analyzed by microRNA microarray. **B:** The expression of the top 6 highly expressed microRNAs (normalized data ≥6) was analyzed by microRNA microarray. **C:** The expression of the 12 screened over-expressed microRNAs was analyzed by qRT-PCR.

**Figure 5:**
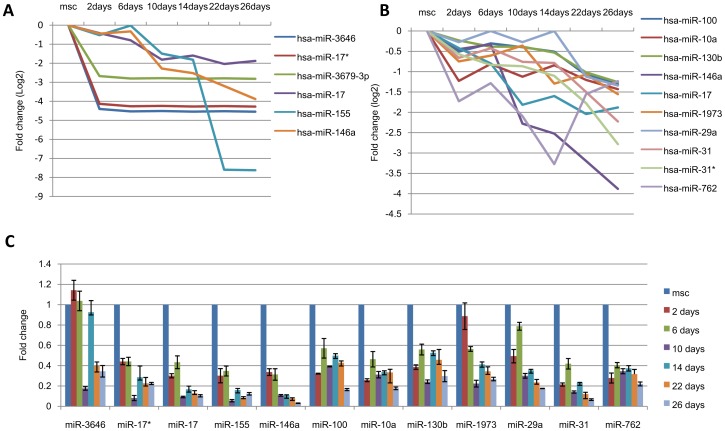
Under-expressed microRNA selection and analysis. **A:** The expression of six microRNAs that changed ≥4-fold was analyzed by microRNA microarray. **B:** The expression of the top 10 highly expressed microRNAs (normalized data ≥6) was analyzed by microRNA microarray. **C:** The expression of the 13 screened under-expressed microRNAs was analyzed by qRT-PCR.

#### Hepatic differentiation

Hepatic differentiation of hUC-MSCs was induced with a Hepatogenic Differentiation Kit, which consists of Pretreatment Medium, Differentiation Medium and Maturation Medium (Cyagen Bioscience). HUC-MSCs were replanted in growth medium at 1×10^4^ cells/cm^2^ in 6-well tissue culture plates pre-coated with FN. After 24 hours, the growth medium was carefully aspirated, and 2 ml Pretreatment Medium was added. After 2days, the medium was changed to Hepatogenic Differentiation Medium by completely replacing the spent pretreatment medium. The cells were re-fed every 3 days for 21 days by completely replacing the medium with fresh Hepatogenic Differentiation Medium. Finally, the medium was replaced with fresh Maturation medium.

**Figure 6:**
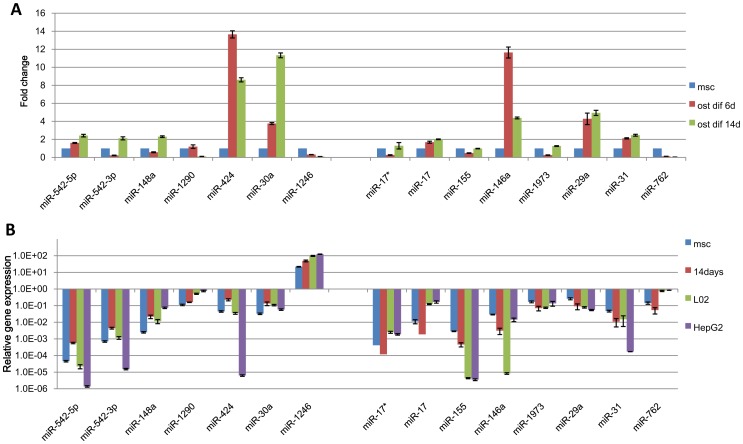
Expression of hepatic differentiated hUC-MSC microRNA profile in osteogenic differentiated hUC-MSC and hepatocyte and hepatocellular carcinoma cells. **A:** MicroRNA expression was analyzed by qRT-PCR at osteogenic differentiated day 6 and day 14 and normalized to U6B expression. Results are expressed relative to a value of 1 in the control hUC-MSCs. **B:** MicroRNA expression was analyzed by qRT-PCR in hepatocyte and hepatocellular carcinoma cells and normalized to U6B expression.

### Quantitative Reverse Transcription-polymerase Chain Reaction (qRT-PCR)

The total RNA from hUC-MSCs obtained at different differentiation time points was isolated with Trizol (Invitrogen, Inc., Carlsbad, CA, USA). A total of 300 ng total RNA was used for cDNA synthesis with the PrimeScript RT Reagent Kit Perfect Real Time (TaKaRa Biotechnology Co., Ltd., Dalian, China). PCR amplification was performed with SYBR Premix Ex Taq TM II (TaKaRa Biotechnology) at 95°C for 30 sec, followed by 45 cycles at 95°C for 15 seconds and 60°C for 20 sec in a light cycler real-time PCR system (Roche Diagnostics)with SYBR green. For each sample, GAPDH expression was analyzed to normalize target gene expression.

**Table 2:** Liver-specific transcription factors and liver-enriched genes that are potentially targeted by altered microRNAs as predicted by TargetScan 6.0.

**microRNAs**	**liver-specific transcription factor**	**liver-enriched gene**
**has-miR-3646**	CRTC2; FOXA1; HNF4A(HNF4); NF1; POU2F1;	APOB; ACACA; PFKFB3;
	SP1;GATA4; NR5A2; STAT1;HNF4G	MET; HNF4A; NR5A2; APP
**has-miR-3679-3p**	CEBPA; CRTC2; CTF; CTF1; NF1; RELA;	ALDOB; PFKFB3; CEBPA;
	POU2F1; SP1;USF1; RXRA; NR5A2; STAT1	MET; NR5A2
**has-miR-155**	CEBPB; FOS; CRTC2; RELA; POU2F1; SP1;	LPL; CETP; R5A2
	RXRA;NR1H3; NR5A2; STAT1	
**has-miR-17**	CRTC2; FOXA1; POU2F1; STAT3; GATA6;	PFKFB3; DLR; PP
	RXRG;HNF4G	
**has-miR-31**	POU2F1; SP1; FOXM1; RXRB; NR5A2	PFKFB3;GYPB;MET; NR5A2; APP
**has-miR-1973**	FOXM1; GATA4	PFKFB3; LDLR
**has-miR-762**	JUN; CEBPA; NR2F1; CTF; HNF1A; HNF4A;	LPL; ACACA; hnf1; PKLR; LDLR;
	NFKB2;RELA; POU2F1; SP1;USF2; RXRB;	CEBPA; MET; HNF4A; srebp2
	RXRA; STAT3;SREBF1(SREBF-1c); SREBF2;	
	GATA4; GATA6; NR1H2	
**has-miR-671-5p**	CEBPA; HNF1A(HNF1); HNF4A; NFKB1;	ACACA; hnf1; LDLR; CEBPA; HNF4A
	POU2F1; SP1;FOXM1; RXRA; STAT3; SREBF1	
**has-miR-542-5p**	NF1; STAT1	PKLR; MET
**has-miR-1185**	CRTC2; POU2F1; USF2; FOXM1; NR5A2	LPL; LDLR; NR5A2; SAA2
**has-miR-539**	CRTC2; FOXA1; NFKB1(NFKB); POU2F1; SP1;	LDLR; HNF4A; srebp2
	RXRB;RXRA; STAT3; SREBF2; GATA4; GATA6;	
	Onecut1A;HNF4G	
**has-miR-542-3p**	JUN(CJUN); CRTC2; HNF1A; HNF4A; RXRB;	hnf1; LDLR; MET; HNF4A; srebp2;
	RXRA;SREBF2; GATA4; NR5A2	NR5A2
**has-miR-1290**	FOXA1; NF1; POU2F1; SP1; GATA4	FOXA2; MET
**has-miR-136**	CRTC2; HNF1A; NF1; POU2F1; SP1; HNF1B;	apob; PL; hnf1; PKLR; CETP; NOS2;
	GATA6;NR5A2	NR5A2; SAA2
**has-miR-424**	CTF; HNF1A; HNF4A; NFKB1; NF1; POU2F1;	APOC3; ACACA; ALDOB; hnf1; NOS2;
	GATA4	HNF4A; APP
**has-miR-1246**	NF1; SP1; RXRA; NR5A2; Onecut1A; FOXM1B	FOXM1B; NR5A2; SAA2

For microRNA analysis, RNA was isolated with Trizol (Invitrogen, Inc., Carlsbad, CA, USA) as described above. cDNA was synthesized with the One Step PrimeScript miRNA cDNA Synthesis Kit Perfect Real Time (TaKaRa Biotechnology). PCR amplification was performed as before. Human U6B was used to normalize target microRNA expression. The primers for qRT-PCR are shown in Table 1. In all of the microRNA analyses, Uni-miR qPCR primer was used as the reverse primer (TaKaRa Biotechnology). Relative changes in gene and miRNA expression were determined with the 2-ΔΔCt method.

### Immunofluorescence

For immunofluorescence staining, cells were fixed with 4% paraformaldehyde for 15 min at room temperature, then incubated with PBS containing 0.2% Triton X-100 for 15 min. The cells were then washed three times with PBS. After blocking with 3% BSA in PBS for 60 min at room temperature, the cells were incubated with primary antibodies at 4°C overnight, washed three times with PBS, and incubated with the appropriate fluorescence-conjugated secondary antibody for 60 min at room temperature in the dark. Nuclei were stained with DAPI (Sigma-Aldrich). Primary and secondary antibodies were diluted in PBS containing 3% BSA. The antibodies used for immunofluorescence are as follows: human anti-ALB (Santa Cruz Biotechnology, California, USA), human anti-CK18 (Santa Cruz Biotechnology), human anti-AFP (Santa Cruz Biotechnology), and Cy5-conjugated goat anti-human IgG (Jackson Laboratories).

### Periodic Acid–Schiff Staining (PAS staining)

The glycogen storage of hepatic differentiated hUC-MSCs was analyzed with a PAS staining kit (Baso Diagnostics Inc., Zhuhai, China). Cells cultured on 8 mm x 8 mm glass coverslips were fixed with PBS containing 4% paraformaldehyde, incubated for 10 minutes in 1% periodic acid, washed with distilled water, and incubated with Schiff’s reagent for 15 minutes. After a 10 minute wash in tap water, the cells were visualized by light microscopy, and images were acquired. HK2 and hUC-MSCs served as positive and negative controls, respectively.

### Urea Assay

Differentiated and undifferentiated hUC-MSCs were cultured for 24 hours in expansion or differentiation medium in the presence or absence of 10 mmol/l NH4Cl. The supernatants were collected, centrifuged, and stored at –20°C until use. Urea concentrations were measured by a BUN assay (Nanjing Jiancheng Bioengineering Institute, Jiangsu, China) according to the manufacturer’s instructions. Expansion or differentiation medium served as a negative control. Finally, the plates were read at a wavelength of 640 nm in an automatic microplate reader (BIO-RAD 680/Bio-Rad Laboratories, Hercules, California, USA).

### LDL Uptake Assay

The LDL uptake ability of hepatic differentiated hUC-MSCs was assessed by fluorescence microscopy after incubation of the cells with 10 mg/ml acetylated LDL labeled with 1, 19-dioctadecyl-3,3,39,39-tetramethylindo-carbocyanine perchlorate (Dil-Ac-LDL) (Yiyuan Biotechnologies, Guangzhou, China). First, Dil-Ac-LDL was diluted to a concentration of 10 µg/ml in complete growth media. The Dil-Ac-LDL solution was then added to cells and incubated for 4 hours at 37°C. The medium was removed, and the cells were washed with probe-free medium and visualized by fluorescence microscopy.

### Microarray Experiments

Cells samples including hUC-MSCs and hUC-MSCs after hepatic differentiation for 2 days, 6 days, 10 days, 14 days, 22 days and 26 days were collected for microRNA expression analysis with Agilent human miRNA (8*60K) V16.0. Microarray experiments including RNA extraction and purification, RNA labeling, array hybridization and data acquisition were performed at Shanghai Biochip Company according to the protocols for the Agilent miRNA microarray system. Total RNA was extracted and purified with a mirVana™ miRNA Isolation Kit (Ambion, Austin, TX, US) and checked for a RIN number to inspect RNA integration with an Agilent Bioanalyzer 2100 (Agilent Technologies, Santa Clara, CA, US). miRNA molecules in the total RNA were labeled with a miRNA Complete Labeling and Hyb Kit (Agilent technologies). Each slide was hybridized with 100 ng Cy3-labeled RNA with a miRNA Complete Labeling and Hyb Kit (Agilent Technologies) in a hybridization oven (Agilent Technologies) at 55°C, and 20 rpm for 20 hours. After hybridization, the slides were washed in staining dishes (Thermo Shandon, Waltham, MA, US) with a Gene Expression Wash Buffer Kit (Agilent Technologies).

### Data Acquisition

Slides were scanned with an Agilent Microarray Scanner (Agilent Technologies) and Feature Extraction software 10.7 (Agilent Technologies) with default settings. Raw data were normalized by the Quantile algorithm in Gene Spring Software 11.0 (Agilent Technologies).

## Results

### HUC-MSCs can be Successfully Induced Into Hepatocytes in vitro

Isolated hUC-MSCs displayed a typical fibroblast-like appearance as MSCs. Flow cytometry analysis confirmed the MSC signature of hUC-MSCs at P3 with a high expression of CD105. The contamination of the culture with hematopoietic cells and endothelial cells was excluded by the absence of CD34 and CD31. Moreover, hUC-MSCs can differentiate into osteoblast-like cells, thus demonstrating their multipotent differentiated potential as MSCs (data not shown).

The hepatic differentiation efficiency of hUC-MSCs was first evaluated by analyzing hepatocyte-specific gene expression at the mRNA and protein levels. QRT-PCR results demonstrated that the expression of HNF4α, ALB and CK18 was increased 10-fold, 4-fold and 3-fold, respectively, after 1 week of induction and reached the highest level after 26 days (Figure 1A). However, after 26 days of induction, apoptotic hepatic-differentiated hUC-MSCs appeared. The expression of hepatocyte-specific genes in the hepatic differentiated hUC-MSCs was also confirmed by immunofluorescence (Figure 1B).

Hepatic differentiation efficiency was also evaluated at the functional level. PAS staining demonstrated that hUC-MSCs submitted to the hepatic differentiation protocol were able to specifically store glycogen compared with undifferentiated hUC-MSCs after induction for 2 weeks (Figure 2A). The ability of hepatic differentiated hUC-MSCs to produce urea was evaluated by exposing the cells to 10 mmol/l ammonium chloride for 24 hours. The urea production ability of hUC-MSCs increased significantly after induction by hepatic differentiation medium and reached the highest level at day 22. At day 26, the urea production ability of the differentiated hUC-MSCs decreased compared to day 22 (Figure 2C). Moreover, after induction for 2 weeks, all hepatic differentiated hUC-MSCs could uptake LDL (Figure 2B). These results indicated that hUC-MSCs differentiated into cells with significant hepatic gene expression and hepatic functions.

### A specific MicroRNA Profile was Expressed during Hepatic Differentiation of hUC-MSCs

At seven time points (2 days, 6 days, 10 days, 14 days, 22 days and 26 days) during the hepatic differentiation of hUC-MSCs, cellular total RNA was collected, including from undifferentiated hUC-MSCs, for microRNA microarray analysis. A total of 1205 human and 144 human viral microRNAs were analyzed. The normalized data demonstrated that 61 microRNAs displayed consistent changes and were altered at least 2-fold between hUC-MSCs and hepatic differentiated hUC-MSCs. Among these microRNAs, 25 were over-expressed; this over-expression occurred either gradually or increased sharply and was maintained at a high level. A total of 36 microRNAs were under-expressed, with an expression pattern similar to that of the over-expressed microRNAs (Figure 3).

To validate the microarray data and screen the microRNAs that are most likely involved in the hepatic differentiation of hUC-MSCs, we selected over-expressed microRNAs and under-expressed microRNAs based on fold changes and expression level for further qRT-PCR analyses. Seven over-expressed microRNAs that were altered at least four fold, including hsa-miR-671-5p, hsa-miR-542-5p, hsa-miR-542-3p, hsa-miR-1185, hsa-miR-539, hsa-miR-148a and hsa-miR-301a, (Figure 4A) and six over-expressed microRNAs that were highly expressed (normalized data ≥6), including hsa-miR-1290, hsa-miR-136, hsa-miR-424, hsa-miR-30a, hsa-miR-148a and hsa-miR-1246 (Figure 4B), were selected for further qRT-PCR analyses. Six under-expressed microRNAs that were altered at least four fold, including hsa-miR-3646, hsa-miR-17*, hsa-miR-3679-3p, hsa-miR-17, hsa-miR-155, and hsa-miR-146a, (Figure 5A) and ten under-expressed microRNAs that were highly expressed (normalized data ≥6), including hsa-miR-100, hsa-miR-10a, hsa-miR-130b, hsa-miR-146a, hsa-miR-17, hsa-miR-1973, hsa-miR-29a, hsa-miR-31, hsa-miR-31* and hsa-miR-762 (Figure 5B), were selected for further qRT-PCR analyses. The data for miR-3679-3p are not shown because the primer that was designed for hsa-miR-3679-3p failed. The qRT-PCR results demonstrated that the expression patterns of hsa-miR-542-5p, hsa-miR-542-3p, hsa-miR-148a, hsa-miR-1290, hsa-miR-424, hsa-miR-30a and hsa-miR-1246 were consistent with the microarray results (Figure 4C). With the exception of miR-3646, all of the under-expressed microRNAs according to microarray were also decreased when analyzed by qRT-PCR. However, only miR-146a displayed consistent changes (Figure 5C).

### microRNA Profile of Hepatic Differentiation was Different from that of Osteogenic Differentiation and Hepatocyte and Hepatocellular Carcinoma Cells

In order to confirm that the microRNA profile expressed during hepatic differentiation of hUC-MSCs was hepatic differentiation specific, the expression of 7 overexpressed and 8 underexpressed microRNAs in hepatic differentiated hUC-MSC selected by microarray and qRT-PCR was analyzed in osteogenic differentiated hUC-MSC at day 6 and day 14. The qRT-PCR results showed that three overexpressed microRNAs(miR-542-5p, miR-424, miR-30a) in hepatic differentiation was also overexpressed in osteogenic differentiation, one underexpressed microRNAs (miR-762)in hepatic differentiation was also underexpressed in osteogenic differentiation. However, miR-1246 which was overexpressed in hepatic differentiation was underexpressed in osteogenic differentiation. Four microRNAs(miR-17, miR-146a, miR-29a, miR-31) which was underexpressed in hepatic differentiation was overexpressed in osteogenic differentiation (Figure 6A). The results indicated that microRNA profile of hepatic differentiation was different from that of osteogenic differentiation.

To confirm the microRNA profile was hepatic differentiation specific but not hepatocyte specific, we further compared the expression of these microRNAs in hepatic differentiated hUC-MSC with in L02, which is a normal human hepatocyte cell line, and HepG2, which is a hepatocellular carcinoma cell line, cultured with hepatic differentiation medium. The expression of miR-542-5p, miR-542-3p and miR-424 in both L02 and HepG2 cells was even lower than in hUC-MSCs. miR-1290 and miR-1246 were enriched in L02 and HepG2 cells, and their expression in hUC-MSC was gradually increased after induction to a level that was similar to that observed in L02 cells. However, the expression of miR-30a after hepatic differentiation was between hUC-MSCs and L02 or HepG2 cells. The expression of five underexpressed microRNAs including miR-155, miR-146a, miR-1973, miR-29a and miR-31during hepatic differentiation in L02 and HepG2 was lower than in hUC-MSCs. However, the expression of other three underexpressed microRNAs including miR-17*, miR-17 and miR-762 during hepatic differentiation in L02 and HepG2 was higher than in hUC-MSCs. (Figure 6B). Thus, the microRNAs involved in hepatic differentiation do not reflect microRNAs enriched in hepatocytes or hepatocellular carcinoma cells.

### MicroRNAs Involved in the Hepatic Differentiation of hUC-MSCs can Potentially Target Liver-enriched Transcription Factors and Genes

MicroRNAs primarily regulate cell differentiation by directly binding to the 3′UTR of the mRNA of the transcription factor. We performed bioinformatics analyses to identify potential targets with TargetScan 6.0, focusing on liver-specific transcription factors and liver-enriched genes (predefined profiles provided by Match™). Most of the microRNAs can potentially target one or several of the 3′ UTRs of liver-specific transcription factors and liver-enriched gene mRNAs (Table 2).

## Discussion

MSCs are fibroblast-like multipotent stem cells that can differentiate into cell types of mesenchymal origin. Under proper culture conditions in vitro or in vivo, MSCs can transdifferentiate into hepatocytes. Moreover, MSC transplantation can markedly improve the levels of ALB, Total bilirubin, and prothrombintime and the MELD scores of liver cirrhosis patients beginning at 2–3 weeks after transplantation [15]. However, the role of microRNAs in this process remains unknown.

To explore whether microRNAs are involved in the hepatic differentiation of MSC, we used hUC-MSCs as seed cells in this study because umbilical cord tissue is an attractive source of MSCs that can be obtained without medical intervention. After successfully inducing hUC-MSC into hepatocytes, we analyzed the expression of 1205 human and 144 human viral microRNAs at seven different time points during the hepatic differentiation of hUC-MSCs. A clearly distinct microRNA microarray profile was observed during the hepatic differentiation of hUC-MSCs. A total 61 microRNAs displayed consistentconsistent changes and were altered at least 2-fold between hUC-MSCs and hepatic differentiated hUC-MSCs, including 25 over-expressed microRNAs and 36 under-expressed microRNAs. The expression of most of these microRNAs was consistent with the results of qRT-PCR analyses.

The microRNAs involved in the hepatic differentiation of hUC-MSCs are different from the microRNAs that participate in hepatocyte regeneration. Several research groups have reported unique microRNA expression profiles in hepatocyte regeneration. Mice with hepatocyte-specific microRNA deficiency were viable and developed normally into adulthood, whereas microRNA-deficient hepatocytes failed to transition into S phase by 36 hours after 2/3 PH. Global microRNA expression analysis demonstrated that the expression of seven microRNAs, including miR-378, miR-689 miR-21, miR-574-5P, miR-696 miR-370 miR-21, that were significantly altered during liver regeneration [16] were not altered during the hepatic differentiation of hUC-MSCs in our study. A total of 26 microRNAs are significantly altered by ≥1.5-fold 3 to 72 h after partial hepatectomy in rats, when critical proliferation and cell division signals are activated, in particular, miR-21. The functional modulation of miR-21 in primary rat hepatocytes can increase cell proliferation and viability [17]. However, there was no overlap between microRNAs in hepatic differentiation and hepatocyte regeneration. miR-34a, which was also highly induced after partial hepatectomy and is up-regulated during liver regeneration in rats and is associated with the suppression of hepatocyte proliferation [18], was not involved in the hepatic differentiation of hUC-MSCs.

The microRNA expression profile during human liver development has also been analyzed [19]. Neither the microRNAs that were over-expressed nor those that were under-expressed during hepatic differentiation of hUC-MSC in our studies overlapped with microRNAs involved in liver development. This may be because prior to birth, the liver is the main site of red blood cell production and liver metabolic functions undergo adaptive changes during ontogeny. The unique microRNA profile mediating hUC-MSC hepatic differentiation was consistent with this differential expression of genes and differential enrichment of transcripts in the fetal and adult mouse liver [20].

However, some of the microRNAs involved in the hepatic differentiation of hUC-MSCs are also involved in the hepatic differentiation of liver-derived progenitor cells (LDPCs).The expression of miR-146a in differentiated LDPCs was highly similar to that in fresh liver cells and significantly lower than that in undifferentiated LDPCs [21]. The expression of miR-542-5p increased in both the hepatic differentiation of hUC-MSCs and LDPCs [21]. MSCs and LDCPs belong to different differentiated and derived adult stem cells, and both can differentiate into hepatocytes. The similarity of the microRNA profiles during hepatic differentiation of LDCPs and hUC-MSCs revealed that their mechanisms may be similar.

As small non-coding RNAs, microRNAs are emerging as key players in the control of cell fate determination at the post-transcriptional level during differentiation. A recent study demonstrated that human fibroblasts can be converted to neurons by overexpressing miR-9/9* and miR-124. The current study provides informative data on changes in microRNAs during the differentiation of hUC-MSCs into hepatocytes and provides the basis for clarifying the role of microRNAs in hUC-MSC hepatic differentiation and specific microRNA selection for the conversion of hUC-MSCs into hepatocytes.

## Supporting Information

Supporting Information S1 **Informed Consent for Umbilical Cord Donation – Chinese.**
(TIF)

Supporting Information S2 **Informed Consent for Umbilical Cord Donation – English.**
(DOC)

Supporting Information S3 **Ethics Statement.**
(TIF)
