# Supplementary figures and images for: Dynamic microRNA Profiles of Hepatic Differentiated Human Umbilical Cord Lining-Derived Mesenchymal Stem Cells
Source: PLoS One. 2012 Sep 12;7(9):e44737. doi: 10.1371/journal.pone.0044737 (PMC3440352; doi:10.1371/journal.pone.0044737)

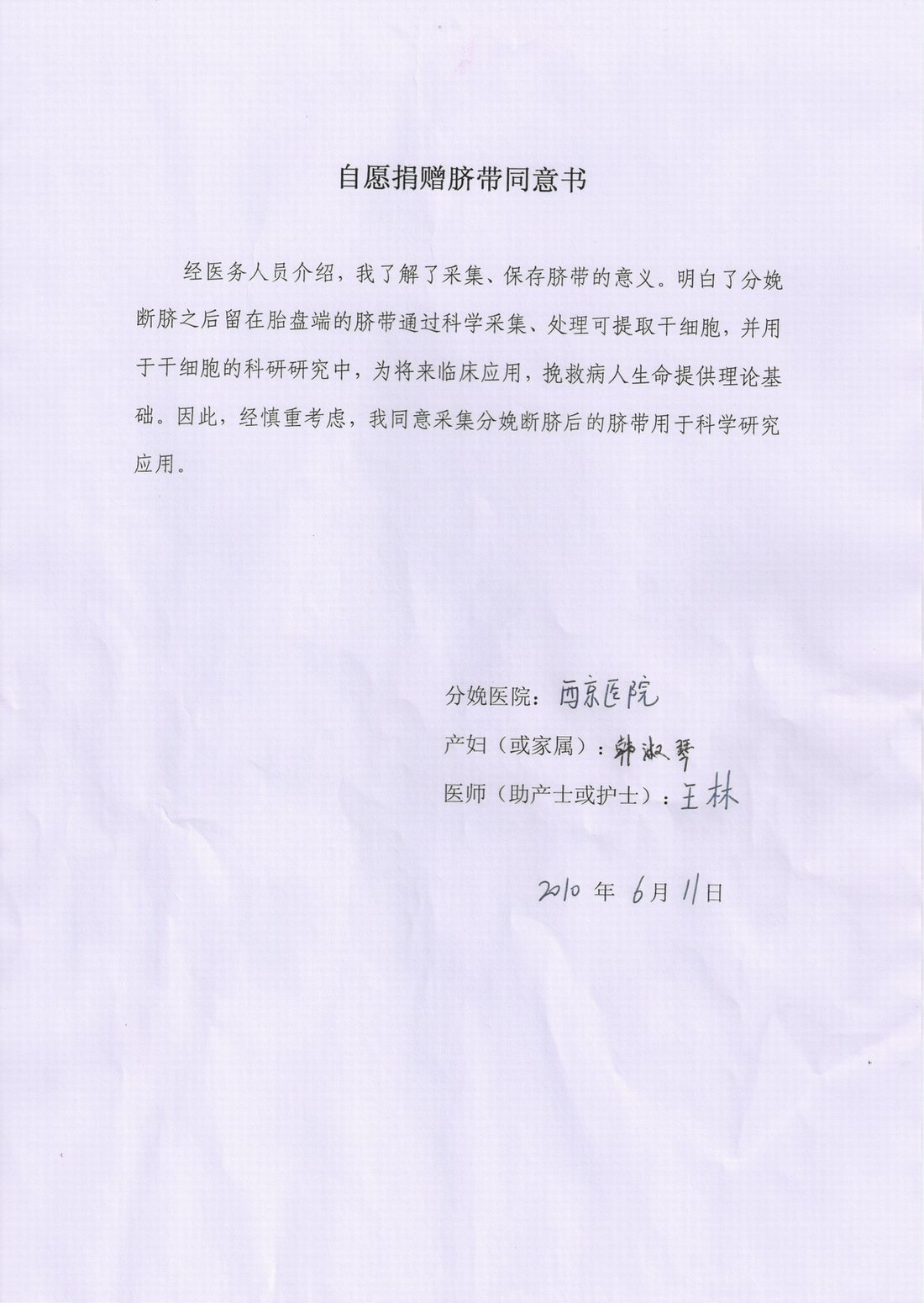

Supplement: Supporting Information S1 — Informed Consent for Umbilical Cord Donation – Chinese. (TIF) [file pone.0044737.s001.tif]

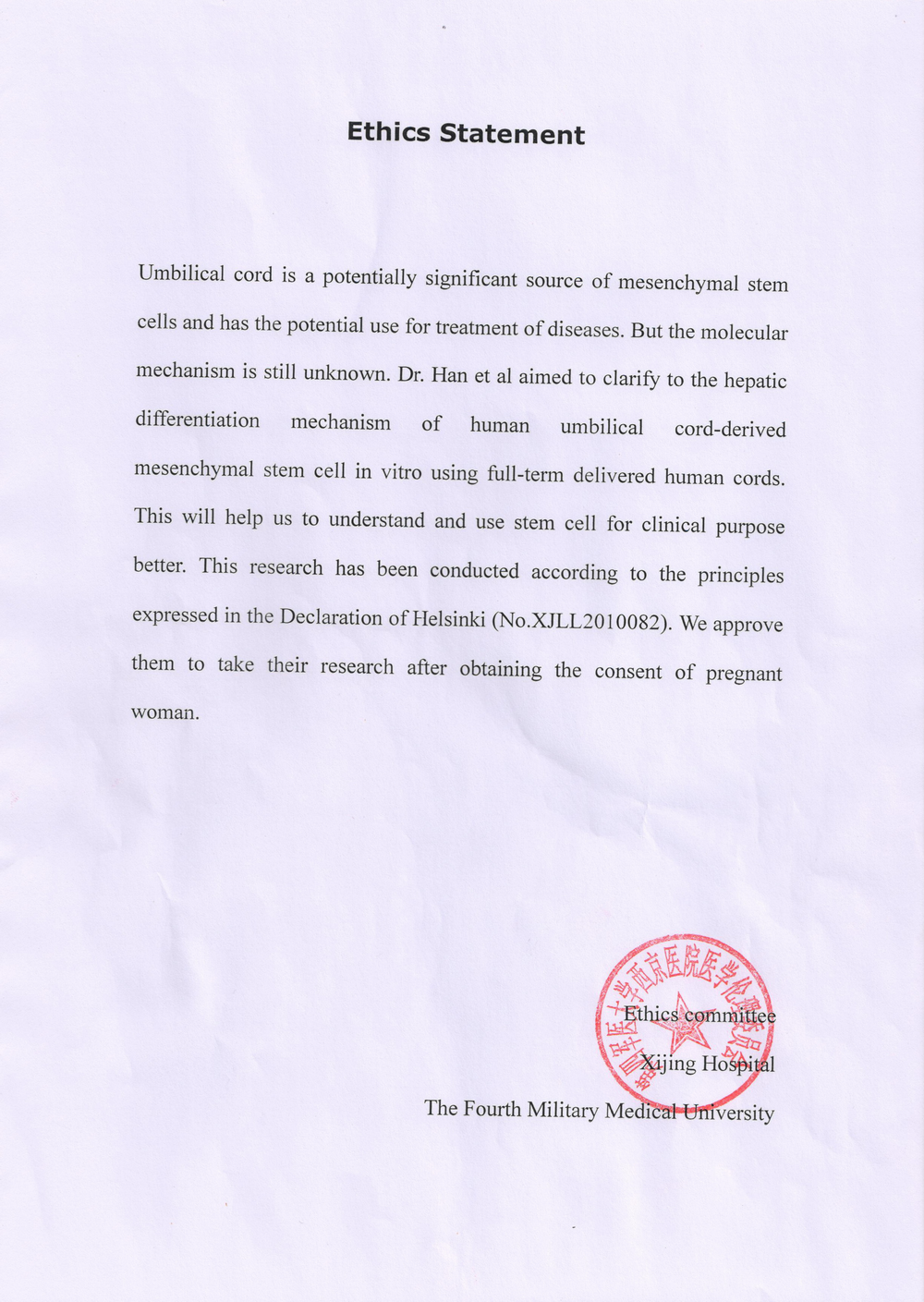

Supplement: Supporting Information S3 — Ethics Statement. (TIF) [file pone.0044737.s003.tif]
