# Supplementary material for: Dynamic microRNA Profiles of Hepatic Differentiated Human Umbilical Cord Lining-Derived Mesenchymal Stem Cells
Source: PLoS One. 2012 Sep 12;7(9):e44737. doi: 10.1371/journal.pone.0044737 (PMC3440352; doi:10.1371/journal.pone.0044737)
Supplement: Supporting Information S2 — Informed Consent for Umbilical Cord Donation – English. (DOC) [file pone.0044737.s002.doc]

**Informed Consent for Umbilical Cord Donation**

After the explanation of doctors, we known that umbilical cord is a potentially significant source of mesenchymal stem cells and have the potential use for clinical and research purposes. In order to improve the use value of umbilical cord, we would like to give our consent of using umbilical cord for research purpose after deliveries.

Hospital：Xijing Hospital

Pregnant woman：Shuqin Han

Doctor：Lin Wang

Date: 2010 Jun 11
